# A PolSAR Image Segmentation Algorithm Based on Scattering Characteristics and the Revised Wishart Distance

**DOI:** 10.3390/s18072262

**Published:** 2018-07-13

**Authors:** Huiguo Yi, Jie Yang, Pingxiang Li, Lei Shi, Fengkai Lang

**Affiliations:** 1 State Key Laboratory of Information Engineering in Surveying, Mapping and Remote Sensing, Wuhan University, Wuhan 430079, China; yihuiguo@whu.edu.cn (H.Y.); pxli@whu.edu.cn (P.L.); shi.lei@whu.edu.cn (L.S.); 2 School of Environment Science and Spatial Informatics, China University of Mining and Technology, Xuzhou 221116, China; langfk@cumt.edu.cn

**Keywords:** PolSAR, segmentation, HLT statistic, scattering characteristics, revised Wishart distance

## Abstract

A novel segmentation algorithm for polarimetric synthetic aperture radar (PolSAR) images is proposed in this paper. The method is composed of two essential components: a merging order and a merging predicate. The similarity measured by the complex-kind Hotelling–Lawley trace (HLT) statistic is used to decide the merging order. The merging predicate is determined by the scattering characteristics and the revised Wishart distance between adjacent pixels, which can greatly improve the performance in speckle suppression and detail preservation. A postprocessing step is applied to obtain a satisfactory result after the merging operation. The decomposition and merging processes are iteratively executed until the termination criterion is met. The superiority of the proposed method was verified with experiments on two RADARSAT-2 PolSAR images and a Gaofen-3 PolSAR image, which demonstrated that the proposed method can obtain more accurate segmentation results and shows a better performance in speckle suppression and detail preservation than the other algorithms.

## 1. Introduction

Synthetic aperture radar (SAR) systems conduct remote sensing and global Earth monitoring under the illumination of radar beams, which offer a day-and-night and all-weather monitoring capability compared with optical sensors. Polarimetric SAR (PolSAR) is the advanced form of SAR, and PolSAR imagery can provide useful information for a diverse number of applications, from target detection [1,2] and sea ice monitoring [3,4] to feature classification [5], agricultural crop identification [6], and geophysical parameter estimation [7,8,9,10]. Gaofen-3 (GF-3) is China’s first fully polarimetric SAR satellite, which was launched on 10 August 2016 from Taiyuan (Shanxi province, China) [11]. GF-3 carries a C-band SAR sensor with 12 different imaging modes [12], which is the largest number of imaging modes of any SAR sensor, and operates in different polarizations, including single-, dual-, and quad-polarizations [13,14]. GF-3 can provide a spatial resolution ranging from 1 m to 500 m and a swath coverage ranging from 10 km up to 650 km [15]. From January 2017, GF-3 began to provide customers with advanced spaceborne SAR imagery which has a full-polarization mode and a resolution as fine as 1 m in spotlight mode [16]. GF-3 will greatly benefit research into SAR image interpretation in the next few years. However, the speckle noise inherent in PolSAR data complicates the image interpretation and analysis and reduces the effectiveness of the applications. Segmentation can mitigate the effects of speckle noise, and it has been clearly demonstrated [17,18] that large performance improvements can be achieved by first segmenting the image into regions with homogeneous characteristics and then classifying the resulting global regions.

Segmentation of PolSAR images has been an ongoing field of research, and numerous algorithms have been proposed. Stewart et al. [19] defined the likelihood function of SAR image segmentation based on the gamma distribution model and, at the same time, the curvature cost function (similar to the surface tension) was introduced to constrain the shape of the segmented region, and thus an appropriate objective function for SAR segmentation was constructed. Dong et al. [20] proposed to use a Gaussian Markov random field (GMRF) model to segment PolSAR intensity images, but in order to simplify the method, the Gaussian distribution was used to replace the gamma distribution, and only the intensity information was used. In [21], a split-merge test was derived for the segmentation of multifrequency PolSAR images following the maximum-likelihood approach. This approach is especially useful in the extraction of information from urban areas that are characterized by the presence of different spectral and polarimetric characteristics. The segmentation method proposed in [22] is equivalent to region merging based on a likelihood-ratio test with an optimized merging order, where the least different pair of neighboring regions is merged in each step. This shows that image segmentation can be viewed as a likelihood approximation problem, and its adaptation for the segmentation of homogeneous and textured scenes has been shown by experiments. Ayed et al. [23] investigated a level set method for PolSAR image segmentation. This approach consists of minimizing a function containing an original observation term derived from maximum-likelihood approximation and a complex Wishart/Gaussian image representation with a classical boundary length prior. The method has also demonstrated its robustness compared with other recent methods. In [24], a Wishart Markov random field (WMRF) model was proposed, in which the Wishart distribution was combined with Markov random fields (MRF) to segment the PolSAR images. The WMRF model is more consistent with the characteristics of PolSAR data than the Gaussian MRF model, so it provides more effective results. The advantage of the spectral graph partitioning segmentation algorithm for PolSAR data was demonstrated in [25]. A region-based unsupervised algorithm that incorporates region growing and a Markov random field edge strength model was proposed in [26] and designed for PolSAR segmentation. The evaluation showed that it improves the segmentation performance by preserving the segment boundaries that the traditional spatial models smooth over. In [27], the statistical region merging (SRM) segmentation algorithm for optical imagery was introduced to PolSAR imagery, and a preliminary improvement was made to make it more suitable for PolSAR data with multiplicative noise. Qin et al. [28] improved the cluster center initialization step and the postprocessing step and extended the simple linear iterative clustering (SLIC) segmentation algorithm for optical imagery to PolSAR imagery, achieving decent results. A novel segmentation method was proposed in [29], which fuses the Dirichlet process mixture model (DPMM) and a similarity measure scheme into the MRF framework. Experiments on real PolSAR images demonstrated its effectiveness.

In this paper, the similarity measured by the complex-kind Hotelling–Lawley trace (HLT) statistic is used to decide the merging order. The merging predicate consists of two steps: Firstly, we judge whether two adjacent pixels are of the same scattering mechanism according to the merging order. Secondly, we compute the revised Wishart distance between the two adjacent pixels which are of the same scattering mechanism, and if the revised Wishart distance is smaller than the preset threshold, we merge the two adjacent pixels. A postprocessing step is applied to obtain a satisfactory result after the merging operation. The decomposition and merging processes are iteratively executed until the termination criterion is met.

The rest of this paper is structured as follows. Section 2 describes the PolSAR data and the model for the covariance matrix data. In Section 3, the proposed segmentation method is presented. In Section 4, the employed PolSAR images are described and the experimental results are reported. Additional discussions are presented in Section 5. Finally, the conclusions are given in Section 6.

## 2. PolSAR Image Model

Polarimetric radar measures the complex scattering matrix of a medium with quad-polarizations [30]. The scattering matrix in a linear polarization base can be expressed as:

(1) $$S=\left[\begin{array}{cc} S_{hh} & S_{hv}\\ S_{vh} & S_{vv} \end{array}\right]$$

where $S_{hv}$ is the scattering element of the horizontal transmitting and vertical receiving polarizations, and the other three elements are similarly defined. For the reciprocal backscattering case, $S_{hv}=S_{vh}$. The polarimetric scattering information can be represented by a complex vector on a linear basis, as shown in:

(2) $$\Omega ={\left[\begin{array}{ccc} S_{hh} & \sqrt{2}S_{hv} & S_{vv} \end{array}\right]}^{T}$$

where the superscript *T* denotes the matrix transpose operation. The vector Ω is a single-look complex (SLC) format representation of PolSAR data. Single- and dual-channel polarimetric data can be treated in a similar way as subsets of a lesser dimension and most likely with less information. The scattering vectors are transformed into multilook sample covariance matrices in order to reduce the speckle noise at the expense of the spatial resolution. The multilook covariance matrix C can be represented as:

(3) $$C=\frac{1}{L}\overset{N}{\underset{i=1}{\sum }}\Omega_{i}\Omega_{i}^{*T}$$

(4) $$C=\left[\begin{array}{ccc} {\langle |S_{hh}|}^{2}\rangle & \langle \sqrt{2}S_{hh}S_{hv}^{*}\rangle & \langle S_{hh}S_{vv}^{*}\rangle \\ \langle \sqrt{2}S_{hv}S_{hh}^{*}\rangle & \langle 2{\left|S_{hv}\right|}^{2}\rangle & \langle \sqrt{2}S_{hv}S_{vv}^{*}\rangle \\ \langle S_{vv}S_{hh}^{*}\rangle & \langle \sqrt{2}S_{vv}S_{hv}^{*}\rangle & {\langle |S_{vv}|}^{2}\rangle \end{array}\right]$$

where *L* is the nominal number of looks used for averaging, the superscript “∗” denotes the complex conjugate, and · denotes the spatial sample averaging. Hence, after multilooking, each pixel in the image is a realization of the d×d stochastic matrix variable denoted as *C*, and the image is referred to as a multilook complex (MLC) covariance image. The dimension *d* is either 1, 2, or 3 depending on the scattering vector used.

It is commonly assumed that the scattering vector Ω jointly follows a circular complex and multivariate Gaussian distribution [31], denoted as $\Omega \sim N_{d}^{C}\left(0,\Sigma \right)$, with a zero mean vector, a true covariance matrix $\Sigma =E\left\{{\Omega \Omega }^{T}\right\}=E\left\{C\right\}$, and dimension *d*. It follows from the Gaussian assumption that if L≥d and the ${\left\{\Omega_{\iota }\right\}}_{\iota =1}^{L}$ are independent, then the unnormalized sample covariance matrix, defined as W=LC, follows a nonsingular complex Wishart distribution [32,33], denoted as $W_{d}^{C}\left(L,\Sigma \right)$. The probability density function (pdf) of *W* is given as:

(5) $$\mathrm{pw}\left(W\right)=\frac{{\left|W\right|}^{L-d}}{\Gamma_{d}\left(L\right){\left|\Sigma \right|}^{L}}exp\left(-tr\left(\Sigma^{-1}W\right)\right)$$

where tr(·) and |·| denote the trace and determinant operators, respectively, and

(6) $$\Gamma_{d}\left(L\right)=\pi^{\frac{d\left(d-1\right)}{2}}\overset{d}{\underset{i=1}{\prod }}\Gamma \left(L-i+1\right)$$

is the multivariate gamma function of the complex kind [34], while Γ(·) is the Euler gamma function. Due to normalization by *L*, the sample covariance matrix *C* follows a scaled complex Wishart distribution [34], denoted as $sW_{d}^{C}\left(L,\Sigma \right)$, whose pdf is:

(7) $$\mathrm{pc}\left(C\right)=\frac{L^{Ld}{\left|C\right|}^{L-d}}{\Gamma_{d}\left(L\right){\left|\Sigma \right|}^{L}}exp\left(-Ltr\left(\Sigma^{-1}C\right)\right).$$

## 3. The Proposed Method

Two important components constitute the proposed method: the merging order followed to test the merging of regions; and the merging predicate, which is applied to judge whether two adjacent regions should be merged or not.

### 3.1. Details of the Processing Steps

**Figure 1:**
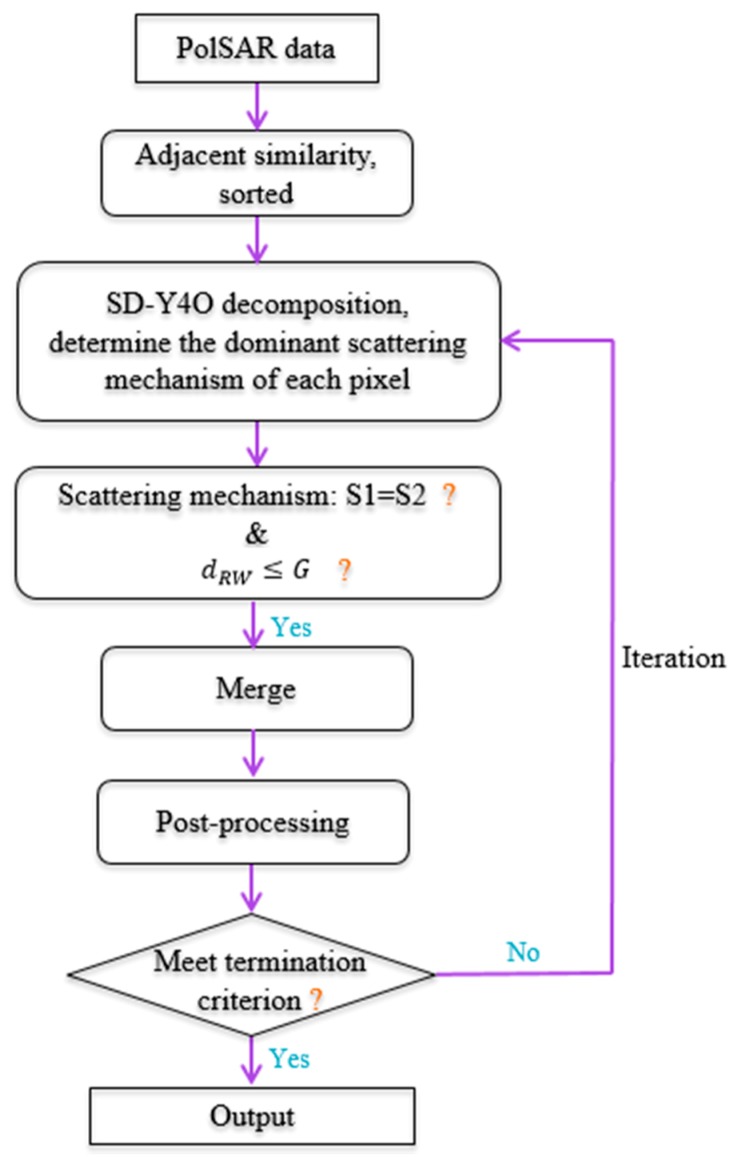
Flowchart of the proposed approach.

**Figure 2:**
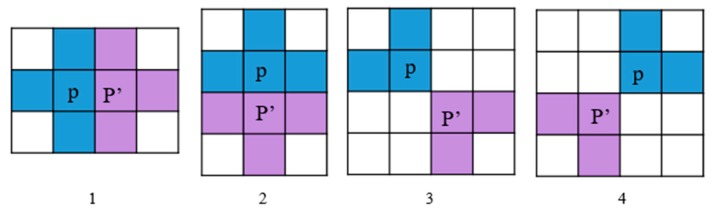
The eight-neighborhood estimation model.

The processing flowchart of the proposed approach is given in Figure 1, and the details of its processing steps are as follows:

1. Compute the similarity of each pixel by Equation (8) according to the eight-neighborhood estimation model in Figure 2. Sort all the similarities in descending order.
2. Apply SD-Y4O decomposition to determine the dominant scattering mechanism of each pixel in the PolSAR image.
3. Calculate the revised Wishart distance of two adjacent pixels which are of the same scattering mechanism according to the descending order of similarities. If the revised Wishart distance is smaller than the threshold set, merge the two adjacent pixels.
4. The postprocessing step is applied after the pixels in the PolSAR image are all processed.
5. After determining the labels of all the pixels, compute the average covariance matrix of the pixels with the same label in the original image, and replace their covariance matrices with the average covariance matrix.
6. The segmented images are iteratively decomposed and merged until the number of pixels whose label changes is less than 5%.

### 3.2. Merging Order

The proposed method calculates the similarity between adjacent pixels according to the eight-neighborhood estimation model [27,35]. In the eight-neighborhood estimation model, p is the central pixel and p′ is the adjacent pixel as shown in Figure 2. The averaged covariance matrix of pixel p is calculated using the blue pixels in the model, and the averaged covariance matrix of pixel p′ is calculated using the purple pixels in the model.

The merging order has a great influence on the merging results, and if the pixels with a higher similarity are merged first, a better segmentation result will be obtained. Therefore, we need to select a simple and efficient method for similarity measurement. This method uses the HLT statistic to measure the similarity between pixel p and pixel p′. The complex-kind HLT statistic is defined as [36]:

(8) $$\tau_{\mathrm{HLT}}=\mathrm{tr}\left(A^{-1}B\right)$$

where A and B are the two PolSAR averaged covariance matrices of pixels p and p′, respectively. In the case of equality of covariance matrices A and B, the value of the test statistic is equal to the polarimetric dimension, i.e., $\tau_{\mathrm{HLT}}=d$. The operator $\tau_{\mathrm{HLT}}$ compacts the matrix-variate quotient into a scalar measure, which can be hypothesis-tested. Calculating the similarity by using the mean value of the pixels surrounding p and p′ is done to reduce the interference of noise and can obtain a more robust similarity value. The HLT statistic is an effective approach for measuring the similarity of two covariance matrices and it also has mathematically simple characteristics.

### 3.3. Merging Predicate

For PolSAR, the scattering characteristics are inherent in the data. These characteristics can provide additional information for the selection of homogeneous pixels. Neighboring pixels, which have similar values in span, may have very different scattering mechanisms that are embedded in the phase differences and correlations between polarizations. Target decomposition can be applied to extract the scattering information, and many different target decomposition methods can be chosen for this purpose [37]. In the proposed method, Bhattacharya decomposition [38] is chosen to decide the dominant scattering mechanism of the pixels. For two adjacent pixels of the same scattering mechanism, we compute the revised Wishart distance [39]. Finally, we merge the two adjacent pixels if the revised Wishart distance is smaller than the threshold.

#### 3.3.1. The Dominant Scattering Mechanisms

Freeman–Durden three-component decomposition can be successfully applied to decompose PolSAR imagery under the well-known reflection symmetry condition using the covariance matrix, but this assumption is often not satisfied in urban areas or other complex areas [40]. Yamaguchi four-component decomposition can be used to deal with the non-reflection symmetric scattering case, where the helix scattering power is added as a fourth component to the three-component scattering model which describes surface, double-bounce, and volume scattering [41]. However, the over-estimation of the volume power and, consequently, the underestimation of the surface and double-bounce powers in the Yamaguchi four-component decomposition model in rotated urban areas is of major concern. The SD-Y4O method [38] estimates the orientation angle from full-polarimetric SAR images using the Hellinger distance. Using this stochastic distance (SD), there is an increase in the surface and double-bounce powers with a corresponding reduction of the volume power. Thus, the surface, double-bounce, and volume powers are systematically modified to obtain appropriate estimates. Therefore, the SD-Y4O decomposition method is utilized to divide the pixels into four dominant scattering mechanisms: surface, double bounce, volume, and helix. The dominant scattering mechanism of each pixel is determined by the maximum in the scattering powers of surface, double-bounce, volume, and helix scattering.

#### 3.3.2. The Revised Wishart Distance

We let $R_{i}$ and $R_{j}$ be the covariance matrix data sets of the *i*th and *j*th regions, respectively, and $\Sigma_{i}$ and $\Sigma_{j}$ are the center covariance matrices of $R_{i}$ and $R_{j}$, respectively. The hypotheses test [42] is:

(9) $$\{\begin{array}{c} H_{0}:\Sigma_{i}=\Sigma_{j}\\ H_{1}:\Sigma_{i}\neq \Sigma_{j} \end{array}.$$

It is assumed that the sample covariance matrices are spatially independent. Therefore, the maximum-likelihood (ML) estimator of $\Sigma_{i}$ is ${\hat{\Sigma }}_{i}=\left(\overset{N_{i}}{\underset{n=1}{\sum }}T_{n}\right)/N_{i}$, and the ML estimator of $\Sigma_{j}$ is ${\hat{\Sigma }}_{j}=\left(\overset{N_{j}}{\underset{n=1}{\sum }}T_{n}\right)/N_{j}$. When $\Sigma_{j}$ is known for hypotheses $H_{0}$ and $H_{1}$, the likelihood-ratio test statistic [28] is:

(10) $$Q=\frac{{\left|{\hat{\Sigma }}_{i}\right|}^{LN_{i}}}{{\left|{\hat{\Sigma }}_{j}\right|}^{LN_{j}}}\exp\left\{-LN_{i}\left(Tr\left({\hat{\Sigma }}_{j}^{-1}{\hat{\Sigma }}_{i}\right)-q\right)\right\}.$$

Thus, the distance measure between the *i*th and *j*th regions becomes the revised Wishart distance:

(11) $$d_{RW}\left(R_{i},R_{j}\right)=-\frac{1}{LN_{i}}\ln Q=\ln\left(\frac{\left|{\hat{\Sigma }}_{j}\right|}{\left|{\hat{\Sigma }}_{i}\right|}\right)+Tr\left({\hat{\Sigma }}_{j}^{-1}{\hat{\Sigma }}_{i}\right)-q.$$

If *i* = *j*, $d_{RW}\left(R_{i},R_{j}\right)$ has a minimum value, i.e., zero; else, the value of $d_{RW}\left(R_{i},R_{j}\right)$ is larger than zero.

### 3.4. Postprocessing

After the pixels in the PolSAR image are all processed, a postprocessing step is applied according to the number of pixels in each region so as to obtain a satisfactory result. We merge the region with its nearest neighbor when its size is less than $N_{min}$. We calculate the dissimilarity between the region and its nearest neighbor when its size is in the range of $\left[N_{min},N_{max}\right]$. If the dissimilarity is smaller than a threshold $G_{th}$, we merge the two regions; else, the region is preserved. The dissimilarity is defined as [28]:

(12) $$G\left(R_{i},R_{j}\right)=\frac{1}{q}{∥\frac{T_{i}^{diag}-T_{j}^{diag}}{T_{i}^{diag}+T_{j}^{diag}}∥}_{1}$$

where $T^{diag}$ denotes the vector composed by the diagonal elements of the central coherence matrix *T* of a region *R*, and $\|.{\|}_{1}$ denotes the 1-norm. Therefore, the range of G is [0,1]. $G_{th}$ was set as 0.3 in all the experiments described in the experiment section of this paper.

## 4. Experiments and Results

To demonstrate the superiority of the proposed approach, we performed segmentation experiments using two RADARSAT-2 PolSAR images and one GF-3 PolSAR image. The proposed method was compared with the conventional mean shift (MS) segmentation method [43], the generalized mean shift (GMS) segmentation method [44], and the generalized statistical region merging (GSRM) method [27].

### 4.1. Evaluation on Two RADARSAT-2 PolSAR Images

The two PolSAR images were acquired by the C-band quad-polarimetric RADARSAT-2 system over the city of Wuhan at two different times. Wuhan, China, which is situated between latitude 29°58′–31°22′ N and longitude 113°41′–115°05′ E, lies in the eastern Jianghan Plain.

#### 4.1.1. Evaluation on the First Data Set

**Figure 3:**
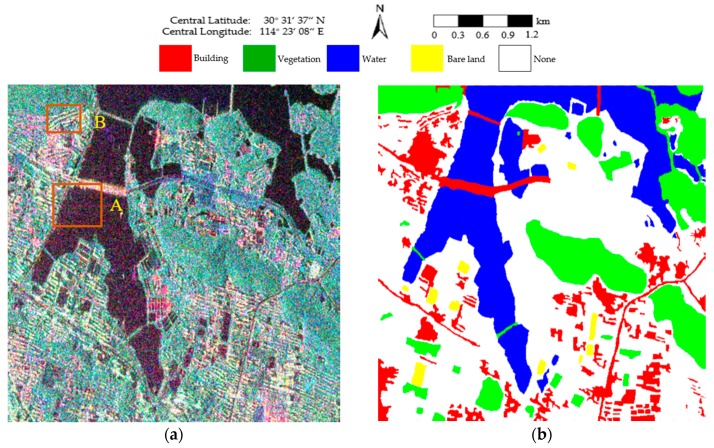
Pauli RGB image and the corresponding ground truth. (**a**) Pauli RGB image of the RADARSAT-2 PolSAR image of size 576 × 579. Areas A and B are enlarged in Figure 6; (**b**) The ground-truth map of (**a**).

The first PolSAR image was collected on 7 December 2011 over the Hongshan District of Wuhan, and has nominal pixel spacings of 4.73 m × 5.12 m (range × azimuth). The experimental image is 576 × 579 pixels in size and is shown in Figure 3a. Four classes, consisting of building, vegetation, water, and bare land, are identified as shown in Figure 3b. The white areas labeled “None” are pixels that are not assigned to any class.

**Figure 4:**
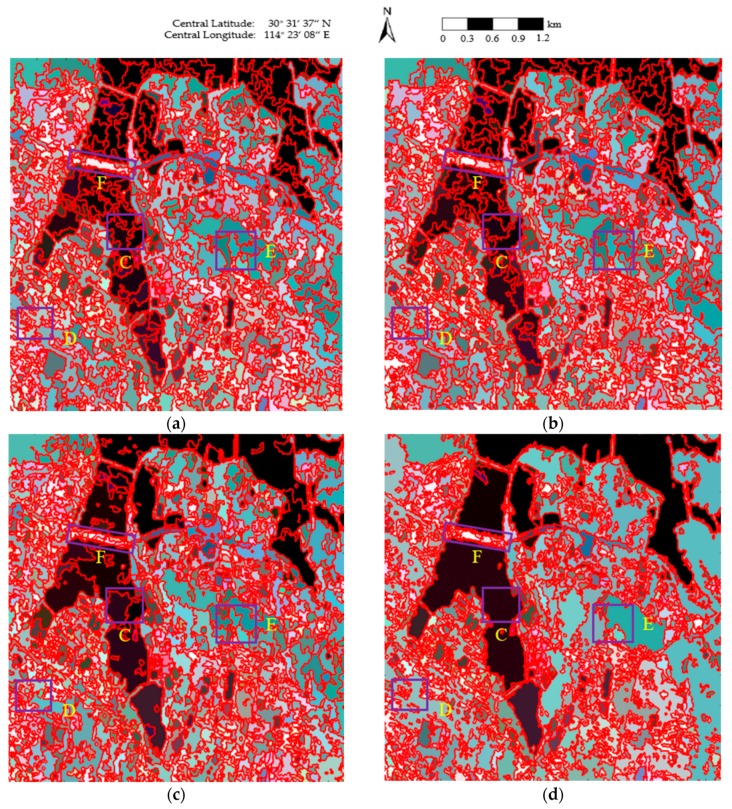
The final maps of the segmentation results obtained using the four methods: (**a**) conventional mean shift (MS); (**b**) generalized mean shift (GMS); (**c**) generalized statistical region merging (GSRM); and (**d**) the proposed method.

The final maps of the segmentation results obtained using the four methods are shown in Figure 4, where the red lines superimposed onto the Pauli RGB images depict the region boundaries. As can be seen in Figure 4a,b, blurred segmentation boundaries are achieved, and a number of small, isolated segments occur in homogeneous areas, such as East Lake (area C of Figure 4a), the urban areas in the Fuhushan Community Neighborhood (area D of Figure 4a), and the forest in Nanwang Mountain (area E of Figure 4a). In Figure 4c, although the region boundaries are not as blurred as in Figure 4a,b, there are still some small, isolated segments in the homogeneous areas, especially in East Lake (area C of Figure 4c). In contrast, much better segmentation results are obtained in Figure 4d. Accurate class boundaries are achieved in areas such as East Lake (area C of Figure 4d), the urban areas in the Fuhushan Community Neighborhood (area D of Figure 4d), the forest in Nanwang Mountain (area E of Figure 4d), and the bridge (area F of Figure 4d). This is because the revised Wishart distance can accurately characterize the similarity between covariance matrices [39,45], which contributes to the precise determination of the homogeneous regions.

**Figure 5:**
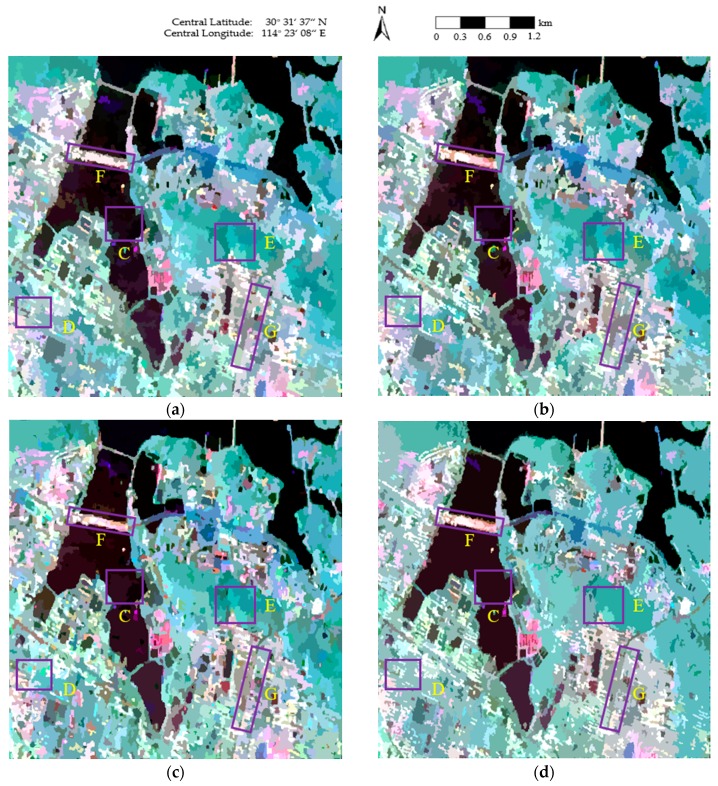
The representation maps of the four methods: (**a**) conventional MS; (**b**) GMS; (**c**) GSRM; and (**d**) the proposed method.

Figure 5 shows the final representation maps, where the covariance of each pixel is replaced by the average value of the region to which the pixel belongs. As can be seen in Figure 5a, the segmentation results are broken, which decreases the visual quality and accuracy of the representation map. In Figure 5b, the roads are well-segmented; however, inaccurate segmentation occurs in areas such as East Lake (area C of Figure 5b), the urban areas in the Fuhushan Community Neighborhood (area D of Figure 5b), and the forest in Nanwang Mountain (area E of Figure 5b). In Figure 5c, the speckle noise is well-suppressed in Nanwang Mountain (area E of Figure 5c), but there are still some areas affected by speckle noise, such as East Lake (area C of Figure 5c). The textures are also not well-maintained in areas such as the urban areas in the Fuhushan Community Neighborhood (area D of Figure 5c) and the roads (area G of Figure 5c). In contrast, the proposed method (Figure 5d) suppresses the influence of speckle noise and provides very smooth approximations in homogeneous areas. The reason for this is that the sufficient homogeneous pixels in the region help to overcome the effect of the speckle noise. The details are also perfectly protected in heterogeneous areas due to the judgment of the same scattering mechanism in the proposed method, which helps to generate accurate segmentation boundaries between the different classes, resulting in precise preservation of feature details.

**Figure 6:**
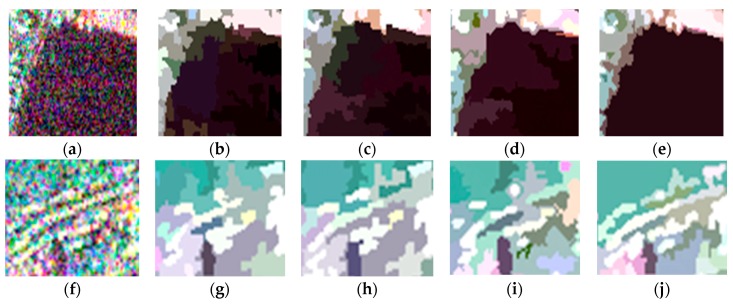
Enlarged versions of areas A and B in the original image and the representation maps obtained using the four methods: (**a**) enlarged version of area A in the original image; (**b**–**e**) enlarged versions of area A in the representation maps obtained using conventional MS, GMS, GSRM, and the proposed method, respectively; (**f**) enlarged version of area B in the original image; (**g**–**j**) enlarged versions of area B in the representation maps obtained using conventional MS, GMS, GSRM, and the proposed method, respectively.

For visual clarity, areas A and B marked by the orange boxes in Figure 3 are enlarged and shown in Figure 6. Of all the boundaries in Figure 6a–e, the boundaries in Figure 6e are smoother and closer to the real terrain edges. In Figure 6f–j, it can be seen that the feature details in Figure 6j are better preserved, which indicates that the proposed method shows a good performance with respect to detail preservation.

To quantitatively evaluate the performance of the four methods, the experimental results were assessed with the commonly used boundary recall (BR) metric [46,47]. BR is the ratio of the boundary pixels shared by the obtained superpixels and the ground truth, which can be represented as:

(13) $$\mathrm{BR}=N_{S\cap G}/N_{G}$$

where $N_{S\cap G}$ denotes the number of superpixels’ boundary pixels overlapping the ground-truth edges, and $N_{G}$ represents the number of ground-truth edges. In this paper, the internal boundaries of the ground truth and the superpixels are employed. In the problem of region generation for PolSAR images, a larger BR value means a better segmentation result.

In Table 1, we list the BR values of the four methods. From Table 1, we can see that the BR value of the proposed method is clearly higher than that of the other segmentation methods. Therefore, we can say that the proposed method obtained more accurate segmentation results than the other methods.

The results of this experiment with the first RADARSAT-2 PolSAR image confirm the effectiveness of the proposed method in PolSAR image segmentation.

#### 4.1.2. Evaluation on the Second Data Set

**Figure 7:**
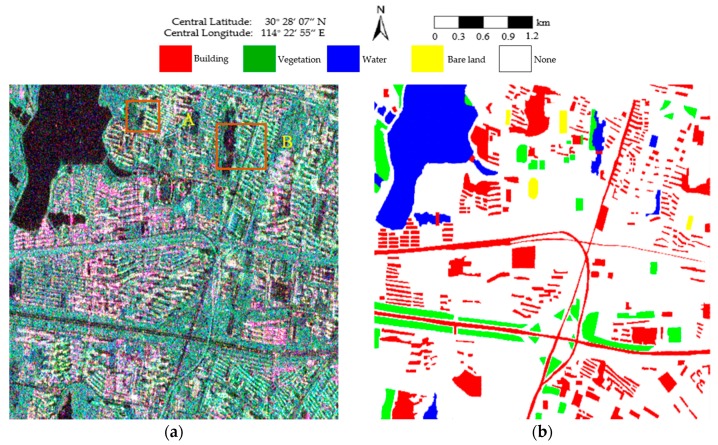
Pauli RGB image and the corresponding ground truth. (**a**) Pauli RGB image of the RADARSAT-2 PolSAR image with a size of 513 × 510. Areas A and B are enlarged in Figure 10; (**b**) The ground-truth map of (**a**).

The second PolSAR data set was collected on 25 June 2015 over the Jiangxia District of Wuhan, with nominal pixel spacings of 4.73 m × 5.12 m (range × azimuth). The experimental image, with a size of 513 × 510 pixels, is shown in Figure 7a. Four classes, consisting of building, vegetation, water, and bare land, are identified as shown in Figure 7b. The white areas labeled “None” are pixels that are not assigned to any class.

**Figure 8:**
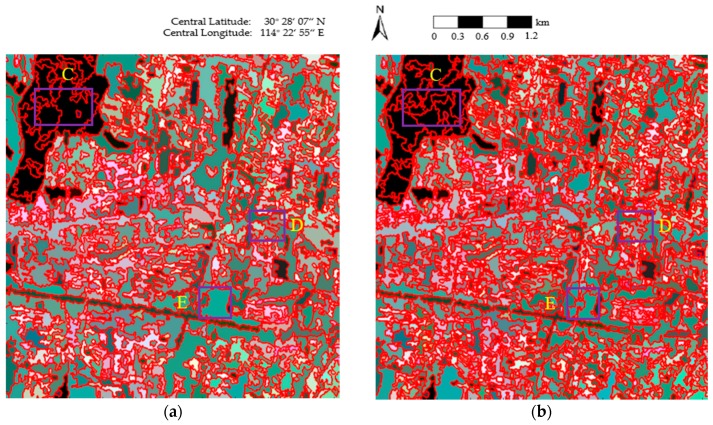

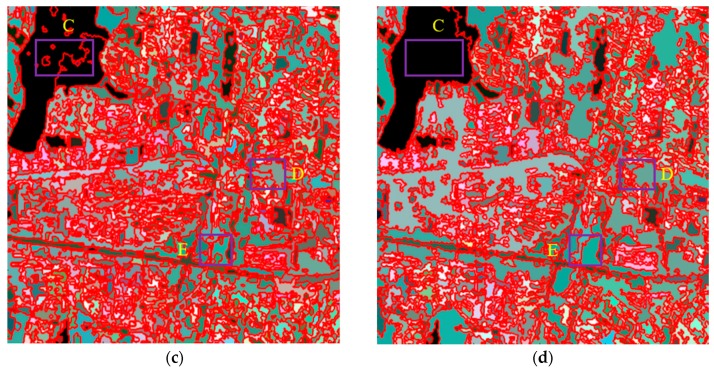
The final maps of the segmentation results obtained using the four methods: (**a**) conventional MS; (**b**) GMS; (**c**) GSRM; and (**d**) the proposed method.

Figure 8 shows the segmentation results of the four algorithms, where the red lines superimposed onto the Pauli RGB images depict the region boundaries. In Figure 8a, there are inaccurate boundaries in homogeneous areas, such as South Lake (area C of Figure 8a) and the urban area in the Dongshangongyu Community Neighborhood (area D of Figure 8a). As can be seen in Figure 8b, there are also numerous inaccurate boundaries occurring in the South Lake area (area C of Figure 8b). In Figure 8c, small, isolated segments occur in the homogeneous areas, such as South Lake (area C of Figure 8c). In Figure 8d, it can be seen that accurate class boundaries are obtained in areas such as South Lake (area C of Figure 8d), the urban area in the Dongshangongyu Community Neighborhood (area D of Figure 8d), and the vegetation area near the Miaoshan overpass (area E of Figure 8d). In the proposed method, the utilization of the revised Wishart distance in the merging predicate is conducive to the accurate generation of homogeneous regions.

**Figure 9:**
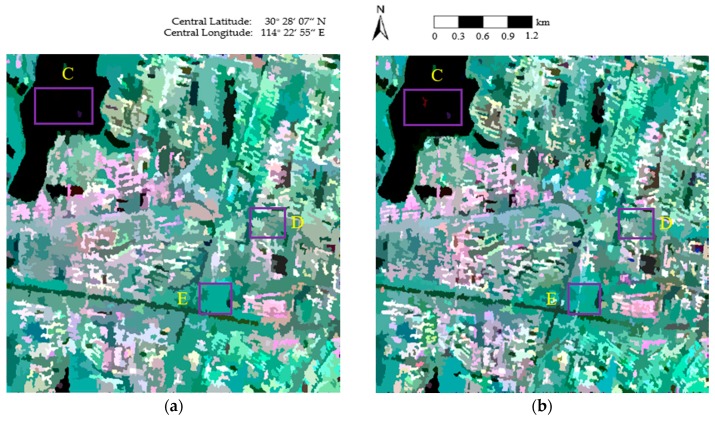

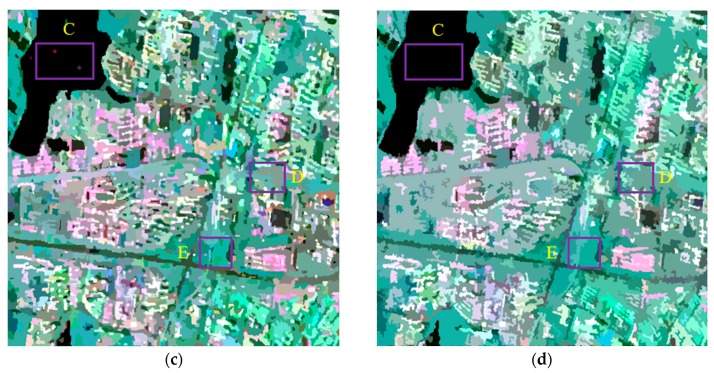
Representation maps of the four methods: (**a**) conventional MS; (**b**) GMS; (**c**) GSRM; and (**d**) the proposed method.

Figure 9 shows the final representation maps, where the covariance of each pixel is replaced by the average value of the region to which the pixel belongs. On the whole, it can be observed that Figure 9d presents a smoother segmentation result, and the speckle noise is well-suppressed in homogeneous areas, such as South Lake (area C of Figure 9d). The textures are also well-maintained in heterogeneous areas, such as the urban area in the Dongshangongyu Community Neighborhood (area D of Figure 9d) and the vegetation area near the Miaoshan overpass (area E of Figure 9d). This is because, in the proposed method, only adjacent pixels of the same scattering mechanism are included in the following merging judgment, which contributes to the precise preservation of feature details.

**Figure 10:**
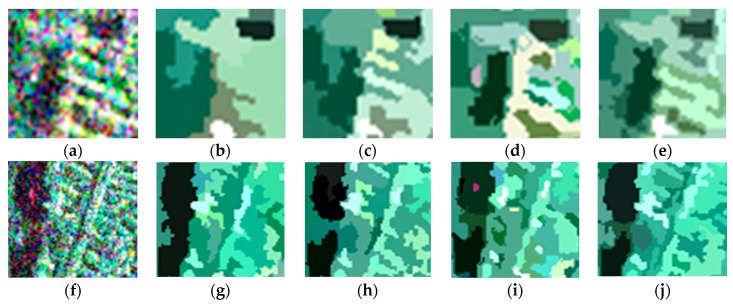
Enlarged versions of areas A and B in the original image and the representation maps obtained using the four methods: (**a**) enlarged version of area A in the original image; (**b**–**e**) enlarged versions of area A in the representation maps obtained using conventional MS, GMS, GSRM, and the proposed method, respectively; (**f**) enlarged version of area B in the original image; (**g**–**j**) enlarged versions of area B in the representation maps obtained using conventional MS, GMS, GSRM, and the proposed method, respectively.

Areas A and B marked by the orange boxes in Figure 7 are enlarged to further illustrate the segmentation effects of the four methods. From all the boundaries shown in Figure 10a–e, it can be clearly observed that the boundaries in Figure 10e are more accurate and adhere better to the real terrain edges. In Figure 10f–j, it can be seen that the feature details in Figure 10j are better preserved, which confirms the good performance in detail preservation of the proposed method.

The BR values of the four methods were calculated to quantitatively evaluate the segmentation performance. As shown in Table 2, it can be seen that the BR value of the proposed method is clearly higher than that of the other segmentation methods, which further demonstrates the advantage of the proposed method.

From the results of this experiment with the second RADARSAT-2 PolSAR image, it is again verified that the proposed method shows an outstanding advantage in PolSAR image segmentation.

### 4.2. Evaluation on a GF-3 PolSAR Image

**Figure 11:**
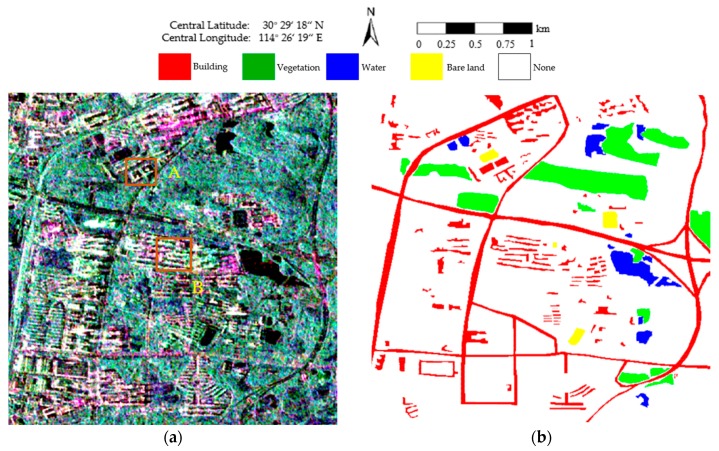
Pauli RGB image and the corresponding ground truth. (**a**) Pauli RGB image of the GF-3 PolSAR image with a size of 519 × 510. Areas A and B are enlarged in Figure 14; (**b**) The ground-truth map of (**a**).

To further validate the effectiveness of the proposed method, we utilized a GF-3 PolSAR image, which was acquired in quad-polarized strip I (QPSI) mode on 30 April 2017 over Wuhan Optics Valley, China, to conduct a segmentation experiment. The experimental image is 519 × 510 pixels in size with a spatial resolution of 8 m as shown in Figure 11a. Four classes, consisting of building, vegetation, water, and bare land, are identified as shown in Figure 11b. The white areas labeled “None” are pixels that are not assigned to any class.

**Figure 12:**
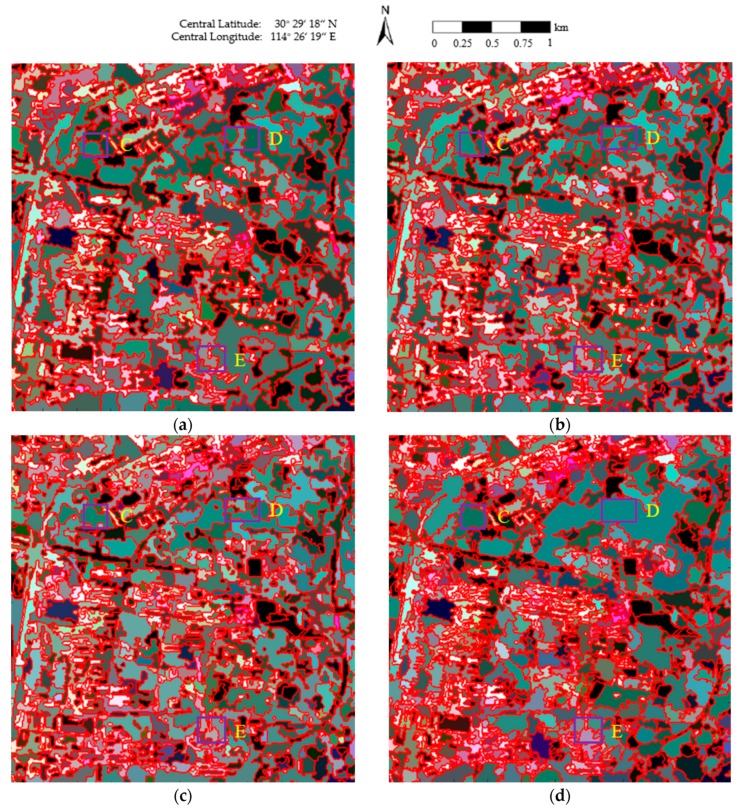
The final maps of the segmentation results obtained using the four methods: (**a**) conventional MS; (**b**) GMS; (**c**) GSRM; and (**d**) the proposed method.

Figure 12 presents the segmentation results of the four algorithms, where the red lines superimposed onto the Pauli RGB images depict the region boundaries. From an overall perspective, the results in Figure 12a–c show clear boundaries. However, the boundaries between the different classes are more accurate in Figure 12d, especially in the forest (area C of Figure 12d), the vegetation area (area D of Figure 12d), and the Jiayuanhuadu Community Neighborhood (area E of Figure 12d). As analyzed above, in the proposed method, the employment of the revised Wishart distance contributes to the precise determination of homogeneous regions.

**Figure 13:**
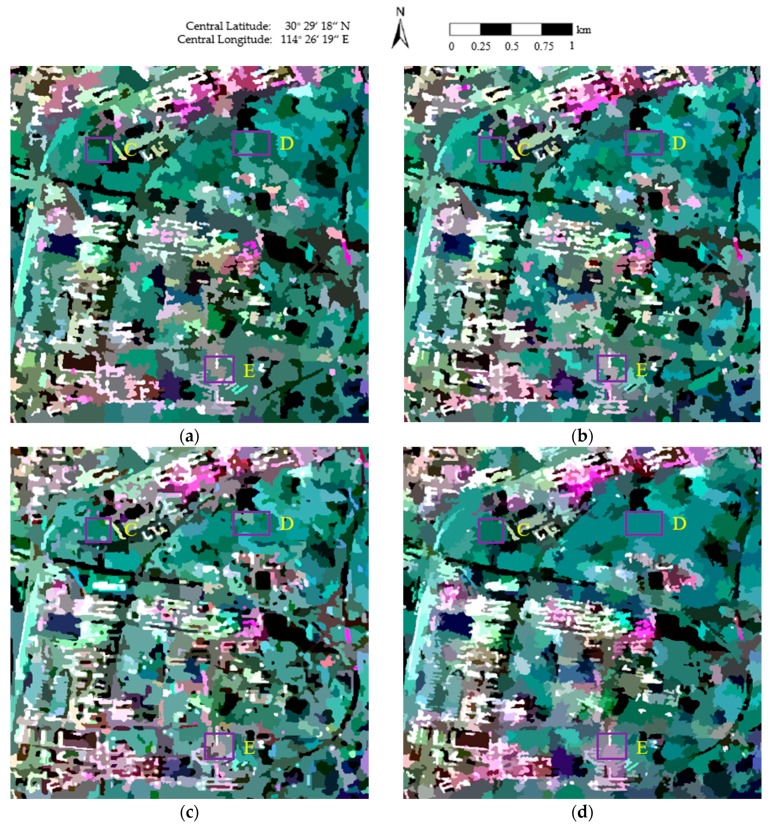
The representation maps of the four methods: (**a**) conventional MS; (**b**) GMS; (**c**) GSRM; and (**d**) the proposed method.

Figure 13 shows the final representation maps, where the covariance of each pixel is replaced by the average value of the region to which the pixel belongs. It can be observed that Figure 13d presents a smoother segmentation result, the speckle noise is well-suppressed in homogeneous areas, and the boundaries are smoother and closer to the real terrain edges in areas such as the forest (area C of Figure 13d) and the vegetation area (area D of Figure 13d). The feature details are also better preserved in heterogeneous areas, such as the Jiayuanhuadu Community Neighborhood (area E of Figure 13d). The reason for this is that the judgment of the same scattering mechanism in the merging predicate is conducive to the accurate preservation of textures.

**Figure 14:**
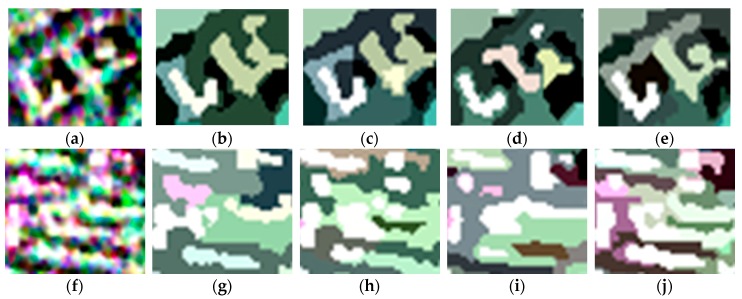
The enlarged versions of areas A and B in the original image and the representation maps obtained using the four methods: (**a**) the enlarged version of area A in the original image; (**b**–**e**) the enlarged versions of area A in the representation maps obtained using conventional MS, GMS, GSRM, and the proposed method, respectively; (**f**) the enlarged version of area B in the original image; (**g**–**j**) the enlarged versions of area B in the representation maps obtained using conventional MS, GMS, GSRM, and the proposed method, respectively.

For visual clarity, areas A and B marked by the orange boxes in Figure 11 are enlarged to further compare the segmentation effects of the four algorithms. From all of the boundaries shown in Figure 14a–e, it can be clearly seen that the boundaries between the different classes in Figure 14e are clearer. In Figure 14f–j, it can be observed that the textures in Figure 14j are better maintained, which confirms the good performance in detail preservation of the proposed method.

To quantitatively assess the segmentation performances, the BR values of the four methods were calculated. As can be seen from Table 3, the BR value of the proposed method is clearly higher than that of the other segmentation methods, which shows that the proposed method can obtain a more accurate segmentation result than the other algorithms.

In summary, the results of this experiment with the GF-3 PolSAR image further confirm the superiority of the proposed method in PolSAR image segmentation.

## 5. Discussion

In this paper, we have proposed a new segmentation method for PolSAR data based on the complex-kind HLT statistic, the scattering characteristics, and the revised Wishart distance. The performance of the proposed method was analyzed using two RADARSAT-2 PolSAR images and a Gaofen-3 PolSAR image, with both visual representation and quantitative evaluation. The experiments and results confirmed the effectiveness and advantage of the proposed method.

Compared with other algorithms, the proposed method has the following advantages:

(1) The revised Wishart distance can accurately characterize the similarity between covariance matrices, which is conducive to the precise determination of homogeneous regions, contributing to the suppression of speckle noise.
(2) The judgment of the same scattering mechanism helps to generate accurate segmentation boundaries between the different classes, resulting in perfect preservation of the feature details.
(3) The SD-Y4O decomposition and merging processes are iteratively executed until the termination criterion is met, which contributes to the accurate segmentation results.

However, there are still some limitations to the proposed method:

(1) The proposed method is implemented by manually setting the revised Wishart distance threshold, and is thus affected by subjective factors to some extent.

## 6. Conclusions

A novel segmentation algorithm for PolSAR images has been presented in this paper. The merging order is based on the similarity measured by the complex-kind HLT statistic. The SD-Y4O decomposition method is applied to divide pixels into the four dominant scattering mechanisms: surface, double bounce, volume, and helix. The merging predicate is determined by the scattering characteristics and the revised Wishart distance between adjacent pixels. A postprocessing step is employed to remove the generated small, isolated regions after the merging operation. The SD-Y4O decomposition and merging processes are iteratively executed until the termination criterion is met. The superiority of the proposed method was verified on two RADARSAT-2 PolSAR images and a Gaofen-3 PolSAR image, with both visual representation and quantitative evaluation. The results demonstrated that the proposed method outperformed the other three methods of conventional MS, GMS, and GSRM in speckle suppression and detail preservation, and could obtain more accurate segmentation results. Nevertheless, the revised Wishart distance threshold needs to be set manually, and is thus affected by subjective factors to some extent. Therefore, in our future work, optimizing the strategy of determining the threshold will help to further improve the performance of the proposed method.

## Figures and Tables

**Table 1:** Boundary recall (BR) values for the first RADARSAT-2 image.

	MS	GMS	GSRM	Proposed Method
BR	0.5441	0.5557	0.5421	0.5871

**Table 2:** BR values for the second RADARSAT-2 image.

	MS	GMS	GSRM	Proposed Method
BR	0.4952	0.5573	0.5472	0.5722

**Table 3:** BR values for the GF-3 PolSAR image.

	MS	GMS	GSRM	Proposed Method
BR	0.4290	0.4532	0.5298	0.5341
